# MIIP accelerates epidermal growth factor receptor protein turnover and attenuates proliferation in non-small cell lung cancer

**DOI:** 10.18632/oncotarget.7001

**Published:** 2016-01-26

**Authors:** Jing Wen, Jianhua Fu, Yihong Ling, Wei Zhang

**Affiliations:** ^1^ Department of Pathology, the University of Texas MD Anderson Cancer Center, Houston, Texas, USA; ^2^ State Key Laboratory of Oncology in South China, Collaborative Innovation Center for Cancer Medicine, Sun Yat-sen University Cancer Center, Guangzhou, Guangdong, P.R. China; ^3^ Department of Thoracic Oncology, Sun Yat-sen University Cancer Center, Guangzhou, Guangdong, P.R. China; ^4^ Department of Pathology, Sun Yat-sen University Cancer Center, Guangzhou, Guangdong, P.R. China

**Keywords:** migration and invasion inhibitory protein, epidermal growth factor receptor, protein degradation, non-small cell lung cancer

## Abstract

The migration and invasion inhibitory protein (MIIP) has been discovered recently to have inhibitory functions in cell proliferation and migration. Overexpression of MIIP reduced the intracellular steady-state level of epidermal growth factor receptor (EGFR) protein in lung cancer cells with no effect on *EGFR* mRNA expression compared to that in the control cells. This MIIP-promoted EGFR protein degradation was reversed by proteasome and lysosome inhibitors, suggesting the involvement of both proteasomal and lysosomal pathways in this degradation. This finding was further validated by pulse-chase experiments using ^35^S-methionine metabolic labeling. We found that MIIP accelerates EGFR protein turnover via proteasomal degradation in the endoplasmic reticulum and then via the lysosomal pathway after its entry into endocytic trafficking. MIIP-stimulated downregulation of EGFR inhibits downstream activation of Ras and blocks the MEK signal transduction pathway, resulting in inhibition of cell proliferation. The negative correlation between MIIP and EGFR protein expression was validated in lung adenocarcinoma samples. Furthermore, the higher MIIP protein expression predicts a better overall survival of Stage IA-IIIA lung adenocarcinoma patients who underwent radical surgery. These findings reveal a new mechanism by which MIIP inhibits cell proliferation.

## INTRODUCTION

Lung cancer is the leading cause of cancer death in both men and women worldwide, accounting for over a million deaths annually [1, 2]. Epidermal growth factor receptor (EGFR), a member of the c-erbB family, is highly expressed in a variety of human tumors, including non-small cell lung cancer (NSCLC), and is implicated in tumor development [3, 4]. EGFR overexpression could be a result of gene amplification, enhanced transcription, or change in protein metabolic turnover [3, 5, 6].

The life of EGFR protein begins at the endoplasmic reticulum (ER). Newly synthesized EGFR peptides insert into the ER, where they undergo N-linked glycosylation co-translationally to form semiglycosylated EGFR [7]. A number of abundant ER residents have been identified as chaperones, such as members of the heat shock protein family, including BIP and its co-chaperone partners. They facilitate protein folding, oligomerization, maturation, and posttranslational modifications. Once the native structures are attained, proteins are released from ER chaperones, and transported from the ER [8]. When exposure to the folding machinery in the ER is not sufficient to promote a native conformation, misfolded proteins are generally degraded by the ER-associated degradation (ERAD) system, a multistep process that targets them to the translocon and retrograde transport back to the cytosol, ubiquitination, and proteasome degradation. Many of the same molecular chaperones involved in folding proteins in the ER are also involved in removal of ERAD substrates, such as BIP [8].

After proper folding in the ER, EGFR protein was transported from the ER to the Golgi apparatus. O-linked oligosaccharides are added to EGFR posttranslationally within the Golgi [7]. Together with the N-linked glycosylation of EGFR in the ER, these modifications confer on EGFR tyrosine kinase autophosphorylation activity [9] and ligand binding activity [10, 11]. The mature EGFR is then transported to the cell membrane.

Once on the membrane, EGF binding to EGFR leads to receptor phosphorylation. The *C*-terminal phosphotyrosine-containing motifs promote receptor ubiquitination. Ubiquitinated EGFR can be recognized by and internalized into clathrin-coated vesicles, and fuse with early endosomes [12]. EGF-EGFR complexes can be rapidly recycled from early endosomes or remain in these endosomes and mature to form multivesicular bodies and late endosomes. Fusion of multivesicular bodies with primary lysosomal vesicles carrying proteolytic enzymes leads to rapid proteolysis of intraluminal components of multivesicular bodies containing EGFR. During its lifetime, an average EGFR will cycle through the endocytic pathway dozens of times. A small increase in the fraction of receptors shunted to the lysosomal pathway can have a large effect on receptor degradation rates.[13].

The balance of these dynamic intracellular trafficking functions (i.e., export, endocytosis, and degradation) dictates the level of receptor expression at the plasma membrane, which in turn influences the magnitude of the cellular response to a given signal.

The migration and invasion inhibitory protein (MIIP), also known as invasion inhibitory protein 45 (IIp45), was found to inhibit cell growth and invasion [14–16]. Our recent research proved that HDAC6, a type II histone deacetylase, is a downstream target of MIIP; MIIP increased HDAC6 protein degradation and enzymatic activity via direct binding [17]. HDAC6 is localized mainly in the cytoplasm and reported to regulates EGFR endocytic trafficking and degradation via deacetylation of α-tubulin [18]. Therefore, we are interested in whether MIIP increases EGFR degradation via HDAC6 or other pathways.

We show here that MIIP expression level correlates negatively with that of steady-state EGFR protein in NSCLC cell lines. In-depth work demonstrated that MIIP increases degradation of immature EGFR protein via a proteasome pathway and of endocytic EGFR via a lysosome pathway, which result in decrease of expression of mature EGFR on the cell membrane, downregulation of the EGFR downstream signaling pathway, and finally reduction of cell proliferation.

## RESULTS

### Effects of altered MIIP expression on steady-state level of EGFR protein in lung cancer cell lines

**Figure 1:**
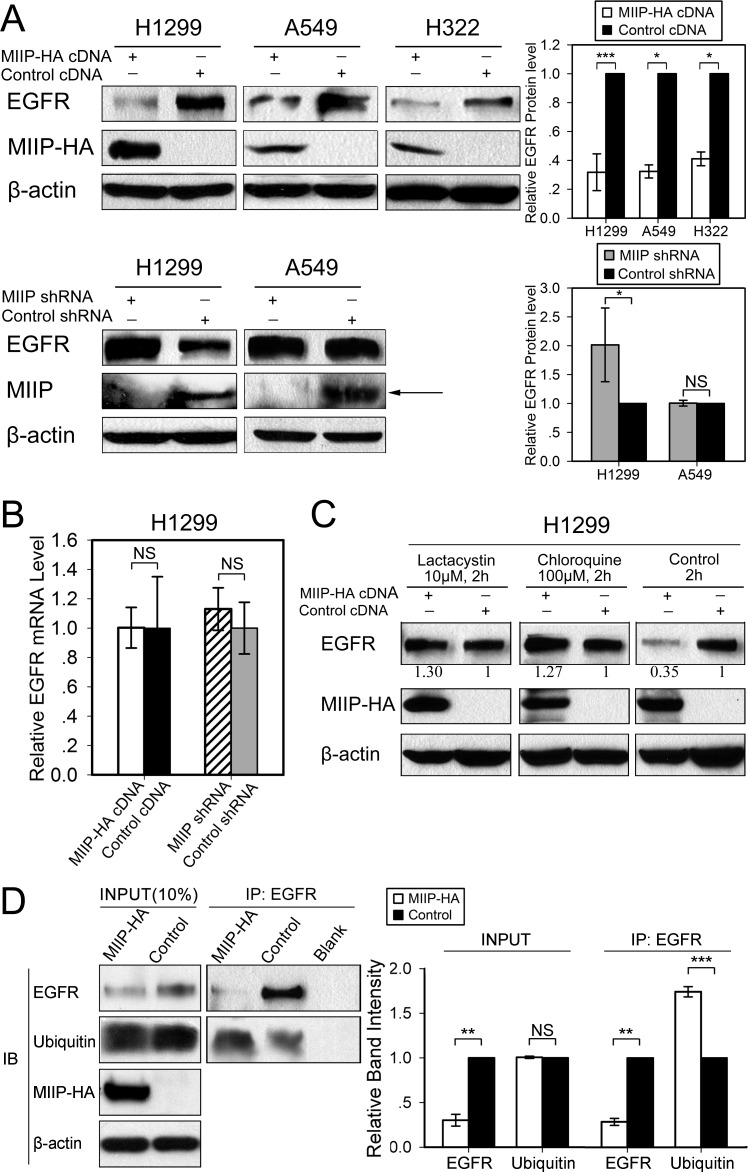
Inverse patterns of MIIP and EGFR protein expression in human lung cancer cell lines **A.** Western blotting analysis of steady-state EGFR protein levels in H1299, A549, and H322 cells transfected with *MIIP*-HA plasmid or with sh*MIIP*. All error bars show standard error for triplicate experiments. *, *P* < 0.05; ***, *P* < 0.001; NS, not significant by Student *t*-test. **B.** Real-time RT-PCR analysis of *EGFR* mRNA levels in MIIP-HA−overexpressing or MIIP-knockdown cells. All error bars show standard error for triplicate experiments. NS, not significant by Student *t*-test. **C.** Western blotting analysis of steady-state EGFR protein levels in MIIP-HA−overexpressing or control H1299 cells with 10 μM lactacystin or 100 μM chloroquine treatment for 2 h. Numbers below the western blotting bands represents the quantification of gel densitometry. **D.** Immunoprecipitation (IP) and immunoblotting (IB) assay of ubiquitinated EGFR in *MIIP*-HA−overexpressing or control H1299 cells with blank protein G beads as negative control. Proteins bound to anti-EGFR-conjugated protein G beads were collected and subjected to IB analysis. INPUT, immunoblot of steady levels of EGFR, ubiquitin, MIIP-HA, and β-actin in cell lysate (10% of the same cell lysate samples used for immunoprecipitation). The intensities of the bands were quantified with densitometry and normalized with that of β-actin loading control. Values are presented in bar graphs as percentage relative to control (100%). All error bars show standard error for triplicate experiments. **, *P* < 0.01; ***, *P* < 0.001; NS, not significant by Student *t*-test.

EGFR protein is widely expressed in lung cancer tissues and most lung cancer cell lines, including H1299, A549, and H322, which were used in our experiments. After transfection with the *MIIP* plasmid, steady-state EGFR protein expression was downregulated to about 30% of that in control cells in the three lung cancer cell lines used (Figure 1A). Knockdown of endogenous MIIP by shRNA in H1299 cells, on the other hand, increased EGFR protein expression significantly (Figure 1A). Interestingly, EGFR protein expression was not increased by *MIIP* shRNA in A549 cells, which had the highest endogenous EGFR levels among the lung cancer cell lines we tested. Other MIIP-independent mechanisms may be critical to maintain such a high level of EGFR in A549 cells. Furthermore, real-time RT-PCR showed no significant alteration in *EGFR* mRNA expression level after MIIP knockdown in H1299 cells (Figure 1B).

### Degradation of EGFR protein promoted by overexpressed MIIP utilizes both proteasomal and lysosomal pathways

Proteasome and lysosome are two major mechanisms adopted by cells to degrade unneeded proteins. We used proteasome inhibitor lactacystin and lysosome inhibitor chloroquine to test whether proteasome and/or lysosome is involved in MIIP-stimulated EGFR degradation. Lactacystin is a highly specific inhibitor of the proteasome [19]. Chloroquine, an antimalarial drug, is a weak base, increasing the lysosomal pH and thereby prohibiting most of the action of lysosomal acid proteinases [20]. Furthermore, chloroquine inhibits transport of hydrolases to the lysosomes and prevents maturation of early endosomes into late endosomes by preventing their acidification. In H1299 cells, either lactacystin or chloroquine reversed MIIP-induced EGFR protein degradation at the steady-state level (Figure 1C), suggesting that both proteasome and lysosome are involved in EGFR degradation in the circumstance of MIIP overexpression. Because ubiquitination of EGFR is critical for its subsequent degradation, we tested whether MIIP might alter EGFR degradation by affecting the levels of ubiquitinated EGFR. The levels of ubiquitinated EGFR were measured in MIIP-overexpression and control H1299 cells. Results showed that MIIP indeed increased the level of ubiquitinated EGFR (Figure 1D).

### Accelerated EGFR protein turnover in MIIP-transfected lung cancer cells

**Figure 2:**
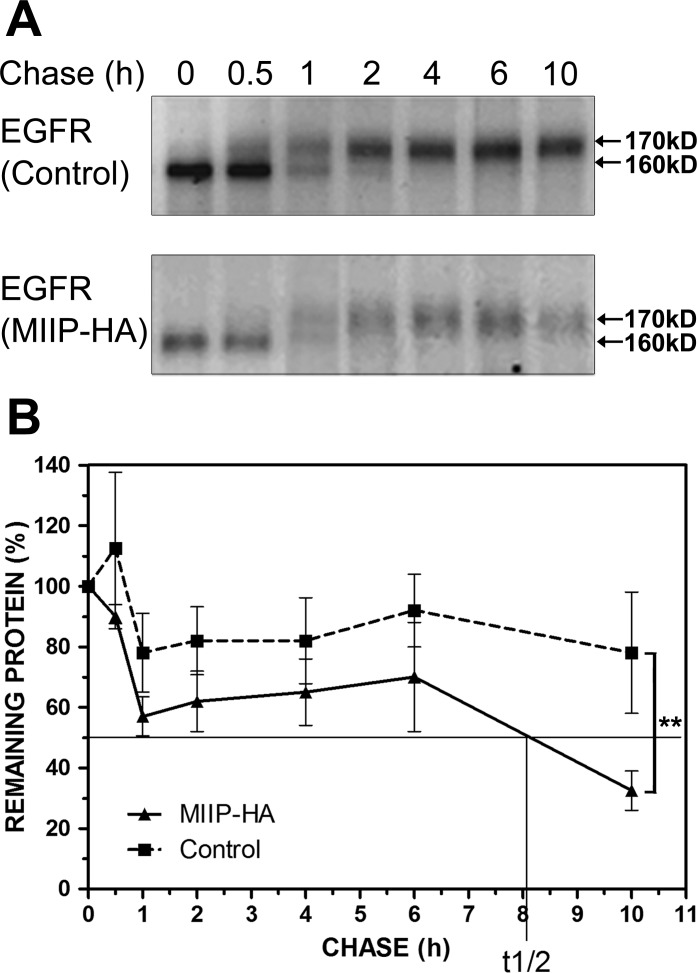
MIIP accelerates EGFR protein turnover Intracellular stability of endogenous EGFR protein level was measured by protein turnover assay in H1299 cells stably transfected with *MIIP* and in control cells. Cells were radiolabeled with ^35^S-methionine in a pulse-chase experiment, and collected at indicated chase time points. Clarified cell lysates were used for immunoprecipitation of endogenous EGFR. Immunoprecipitated proteins were resolved on SDS-PAGE and detected by fluorography. **A.** Increased turnover rate of endogenous EGFR protein was shown in cells stably transfected with *MIIP*. **B.** Graphic representation of EGFR protein turnover is based on quantification of gel densitometry. All error bars show standard error for triplicate experiments. **, *P* < 0.01 by repeated measures ANOVA.

It is possible that MIIP downregulates the intracellular steady-state level of EGFR by destabilizing the endogenous EGFR protein. To determine the effect of MIIP on intracellular stability of EGFR, we examined the half-life of endogenous EGFR protein in *MIIP*-HA−transfected H1299 cells and compared it with that of control cells. Consistently with previous results [21], the newly synthesized endogenous EGFR was stable over 10 h in H1299 control cells. In MIIP-overexpressing cells, however, the half-life of endogenous EGFR was reduced to about 8 h (Figure 2). In other words, EGFR degradation in *MIIP*-transfected cells was accelerated. Figure 2A illustrates the results of pulse-chase experiments that show the turnover of the smaller, core-glycosylated precursor form of EGFR (160 kD), the appearance of its larger, complex-glycosylated product (170 kD), and the turnover of the mature EGFR.

The smaller, immature band disappeared totally after about 4 h of chase in both MIIP-HA−overexpressing and control H1299 cells (no significant difference). This agrees with the findings of an earlier study of EGFR in A431 cells where conversion of the 160-kD EGFR precursor to its 170-kD mature form is a slow process, with a half-time for conversion of approximately 1.7 h [22]. In the control H1299 cells, about 20% of the EGFR precursor was degraded in the first 4-h chase period, during which the semiglycosylated form was fully converted into the mature one. In MIIP-HA−overexpressing cells, however, the turnover of the semiglycosylated precursor band was greatly accelerated, with about 40% degraded in first 4-h chase, although the conversion was not delayed. As 3-4 h are required before maximum labeled receptor is detected on the cell surface [22], MIIP appeared to accelerate degradation of the newly synthesized endogenous EGFR protein before its maturation and transport to the cellular membrane. On the other hand, about 55% of the mature EGFR was degraded during the period from chase 2 h to 10 h in *MIIP*-HA−transfected H1299 cells. Only 5% was degraded during the same period in control cells. (Figure 2B) Therefore, MIIP accelerates degradation of both semiglycosylated and mature EGFR in H1299 lung cancer cells.

### MIIP promotes degradation of newly synthesized EGFR protein by ER-associated proteasomal degradation

**Figure 3:**
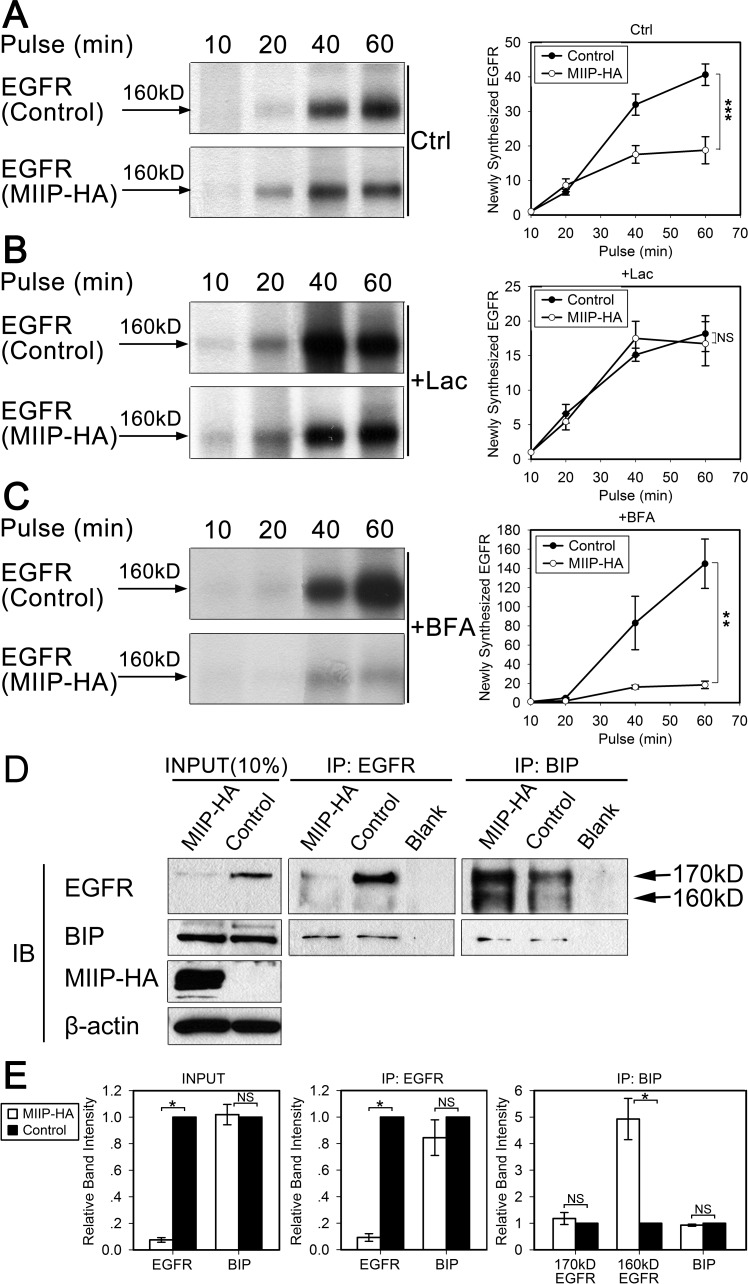
MIIP promotes degradation of newly synthesized EGFR by the proteasome pathway H1299 cells stably transfected with *MIIP* and control cells were radiolabeled with ^35^S-methionine for indicated time in a pulse experiment without drug treatment (Ctrl; A) or with 10 μM lactacystin (Lac; B) or 5 μM brefeldin A (BFA; C) treatment. Clarified cell lysates were used for immunoprecipitation of endogenous EGFR. Graphic representation of EGFR protein turnover is based on quantification of gel densitometry from triplicate experiments. **A.** Turnover of newly synthesized endogenous EGFR in MIIP-HA−overexpressing or control H1299 cells. ***, *P* < 0.001 by repeated measures ANOVA. **B.** Turnover of newly synthesized endogenous EGFR in MIIP-HA−overexpressing and control H1299 cells with lactacystin treatment. NS, not significant by repeated measures ANOVA. **C.** Turnover of newly-synthesized endogenous EGFR in MIIP-HA−overexpressing or control H1299 cells with brefeldin A treatment. **, *P* < 0.01 by repeated measures ANOVA. **D.** Reciprocal co-immunoprecipitation (IP) assay of endogenous EGFR and BIP in MIIP-HA−overexpressing or control H1299 cells with blank protein G beads as negative control. Proteins bound to protein G beads were collected and subjected to SDS-PAGE/western blotting (IB) analysis. INPUT, immunoblot of steady levels of EGFR, BIP, MIIP-HA, and β-actin in cell lysate (10% of the same cell lysate samples used for immunoprecipitation). **E.** The intensities of the bands in *D* were quantified with densitometry and normalized with that of β-actin loading control. Values are presented in bar graphs as percentage relative to control (100%). All error bars show standard error for triplicate experiments. *, *P* < 0.05; NS, not significant by Student *t*-test.

We utilized a pulse experiment without drug or with lactacystin or brefeldin A to determine the subcellular location of immature EGFR degradation and which protein degradation pathway was involved. As shown in Figure 3A, less semiglycosylated EGFR was observed after a 60-min pulse in MIIP-overexpressing H1299 cells than in control cells. This could have been caused by either less protein synthesis or more protein degradation. When proteasome inhibitor lactacystin was added during the pulse, the intensities of the semiglycosylated EGFR bands for the MIIP-overexpressing and control H1299 cells were not significantly different (Figure 3B), suggesting that the lower level of expression of semiglycosylated EGFR observed in MIIP-overexpressing cells was due to accelerated proteasome-mediated protein degradation, not to decreased protein synthesis. Furthermore, brefeldin A, which inhibits transport of proteins from the ER to the Golgi apparatus and induces retrograde protein transport from the Golgi to the ER[23], enhanced degradation of newly synthesized EGFR in MIIP-overexpressing H1299 cells (Figure 3C). Therefore, we assumed that MIIP overexpression accelerates degradation of newly synthesized EGFR protein in the ER.

Since BIP is the most abundant ER chaperone and is involved in exocytic protein folding [8], we investigated BIP expression and its binding with EGFR in MIIP-overexpressing and control H1299 cells. BIP expression was not significantly different between MIIP-overexpressing cells and control cells. However, binding of semiglycosylated EGFR and BIP was increased in MIIP-overexpressing cells (Figure 3D and 3E).

### MIIP accelerates degradation of mature EGFR protein

**Figure 4:**
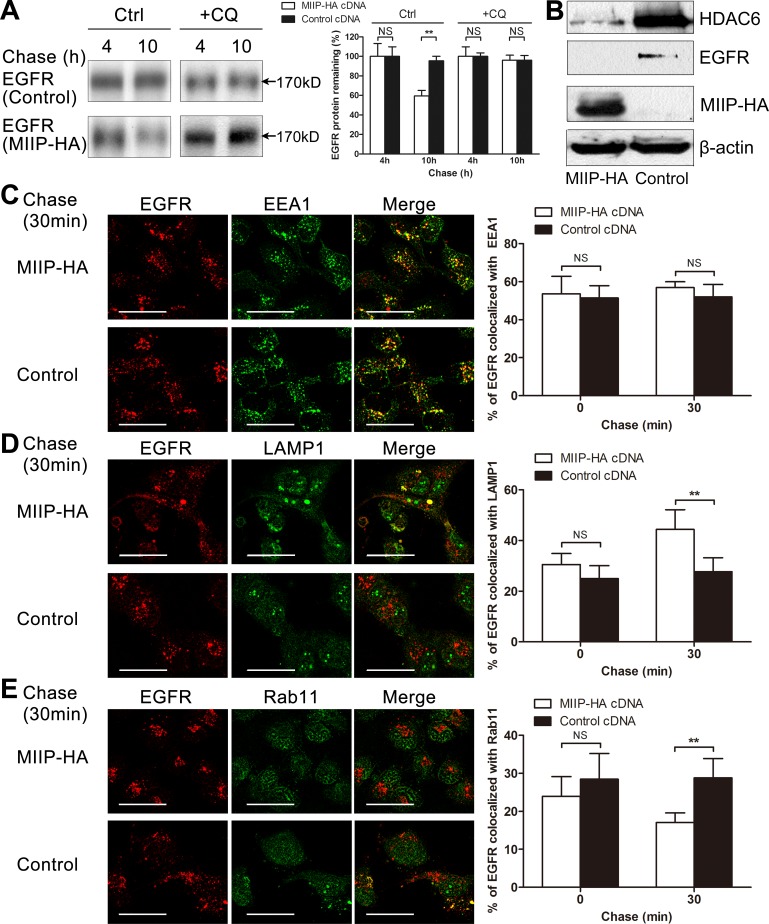
MIIP accelerates lysosomal degradation of mature EGFR **A.** H1299 cells stably transfected with *MIIP*-HA and control plasmid were radiolabeled with ^35^S-methionine for 40 min and chased for indicated time without (Ctrl) or with 100 μM chloroquine (CQ). Graphic representation of EGFR protein turnover is based on quantification of gel densitometry from triplicate experiments. NS, not significant; **, *P* < 0.01 by Student *t*-test. **B.** Western blotting determined expression of steady-state HDAC6 protein in freshly *MIIP*-HA-transfected and control H1299 cells. **C.** H1299 cells stably transfected with *MIIP*-HA and control plasmid were treated with 10ng/ml EGF for 5min, chased for the indicated time, and then subjected to immunofluorescence staining with anti-EGFR (red) and anti-EEA1 (green). Quantification of colocalization between EGFR and EEA1 signals is shown in the histograms. NS, not significant by Student *t*-test. (Scale bar: 40μM) **D.** H1299 cells stably transfected with *MIIP*-HA and control plasmid were treated as described in B and immunofluorescent stained with anti-EGFR (red) and anti-LAMP1 (green). Quantification of colocalization between EGFR and LAMP1 signals is shown in the histograms. NS, not significant; **, *P* < 0.01 by Student *t*-test. (Scale bar: 40μM) **E.** H1299 cells stably transfected with *MIIP*-HA and control plasmid were treated as described in B and immunofluorescent stained with anti-EGFR (red) and anti-Rab11 (green). Quantification of colocalization between EGFR and Rab11 signals is shown in the histograms. NS, not significant; **, *P* < 0.01 by Student *t*-test. (Scale bar: 40μM)

Significant degradation of mature EGFR protein was observed in MIIP-overexpressing cells (Figures 2 and 4A). Membrane-located EGFR is constantly shuttled between membrane and endocytic vesicles. EGFR could be rapidly recycled from endosomes or remain in the endosomes, which are finally degraded in lysosome [13, 24–26]. When lysosome inhibitor chloroquine was added to the medium, degradation of mature EGFR was reversed (Figure 4A), suggesting that lysosome is involved in MIIP-promoted degradation of mature EGFR protein. It was recently reported that HDAC6, one of the downstream targets of MIIP [17], regulates the endocytic trafficking and degradation of EGFR protein by accelerating segregation of EGFR from early endosomes and premature delivery of EGFR to the late endosomal and lysosomal compartments [18]. Since MIIP overexpression decreased HDAC6 expression in H1299 cells (Figure 4B), MIIP might increase EGFR degradation during its endocytosis via downregulation of HDAC6.

Given the evidence that MIIP enhances mature EGFR degradation in lysosome, we next used immunofluorescence microscopy to examine whether MIIP controls receptor trafficking through endo-lysosomal compartments in H1299 cells expressing ectopic MIIP. When cells were treated with EGF for 5min, there were no significant differences of the colocalization of EGFR with the early endosome marker EEA1 (53.6% vs. 51.5%, *P* > 0.05), late endosome and lysosome marker LAMP1 (30.5% vs. 25.0%, *P* > 0.05), and recycling endosome marker Rab11 (24.0% vs. 28.4%, *P* > 0.05) between MIIP-HA−overexpressing and control cells (Figure 4C-4C). Then we chase the cells' endo-lysosomal trafficking of the internalized EGFR by incubating the EGF-treated cells in both EGF- and serum-free medium. After 30min chase, the colocalization of EGFR with EEA1 is comparable in MIIP-HA overexpressing and control H1299 cells (56.9% vs. 52.0%, *P* > 0.05, Figure 4C), indicating that MIIP does not regulated receptor traffic through early endosome. However, increased colocalization of EGFR with LAMP1 (44.4% vs. 27.7%, *P* < 0.01, Figure 4D) but decreased with Rab11 (17.0% vs. 28.8%, *P* < 0.01, Figure 4E) in MIIP-HA−overexpressing H1299 cells was observed in comparison with control cells, suggesting MIIP promoting EGFR trafficking to late endosome or lysosome while decreasing EGFR recycling through recycling endosome.

### Effects of EGFR protein instability on EGF-binding activity, activation of downstream Ras and MEK/ERK signaling, and cell proliferation

EGFR is a transmembrane glycoprotein with an extracellular ligand-binding domain and a cytoplasmic domain with intrinsic tyrosine kinase activity. Upon activation by its growth factor ligands, such as EGF, EGFR undergoes a transition from an inactive monomeric form to an active homodimer or heterodimer, which stimulates its intrinsic intracellular protein-tyrosine kinase activity and leads to autophosphorylation of key tyrosine (Y) residues in the *c*-terminal domain of EGFR and further activation of downstream signaling events. Among the autophosphorylated tyrosine residues, Y1068 is a key site in mediation of downstream Ras activation. The phosphotyrosine 1068 of activated EGFR is bound by the Grb2/Sos complex, which brings Sos into close proximity with Ras and leads to activation of Ras. Once Ras is activated, it proceeds to stimulate a cascade of protein kinases, such as MEK/ERK, which are important in a myriad of growth factor responses [27].

**Figure 5:**
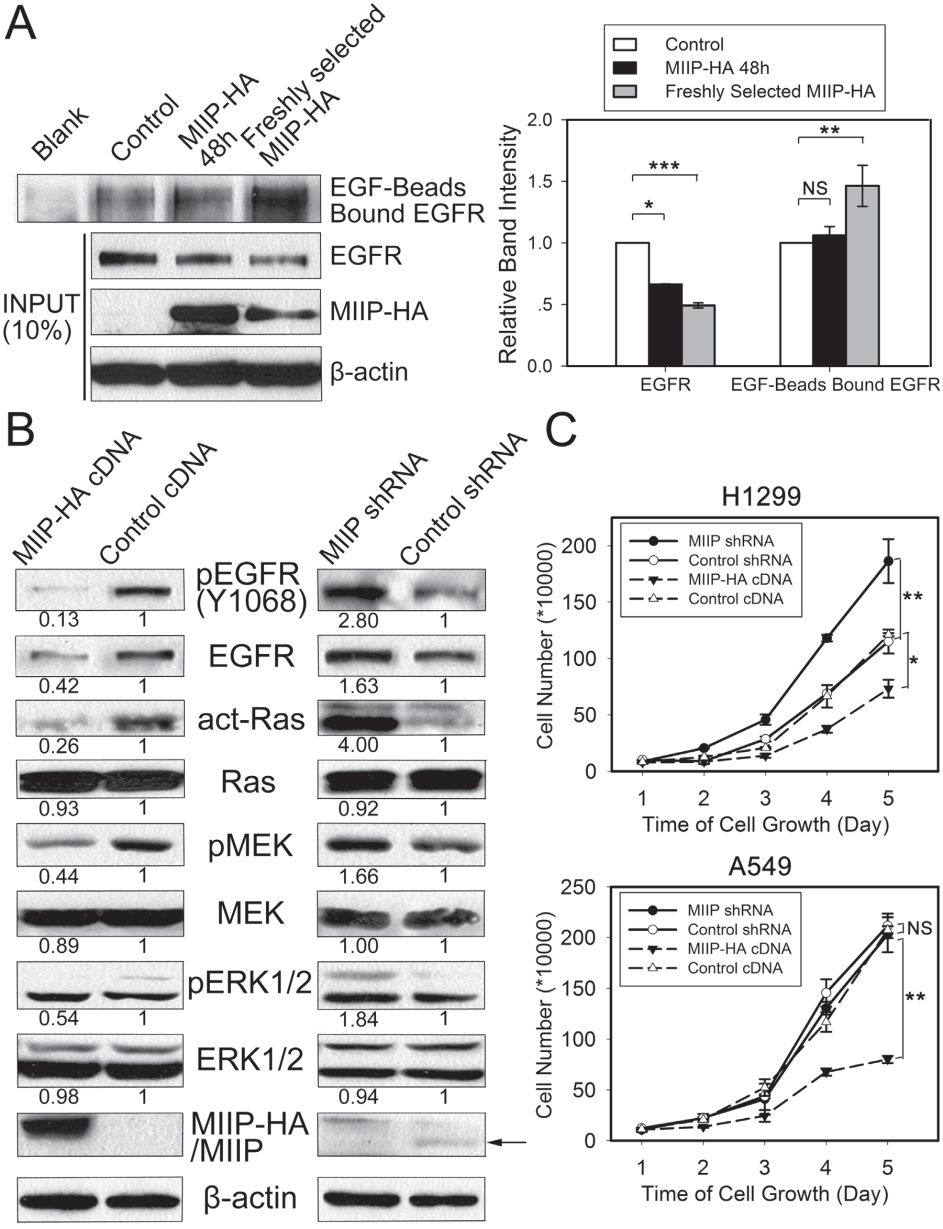
Functional analysis of the effects of MIIP expression on EGFR's EGF-binding activity and activation of EGFR and downstream signaling and cell proliferation **A.** EGF-binding activity was determined in *MIIP*-HA−overexpressing and control H1299 cells. EGFR bound to the EGF-conjugated beads was pulled down and subjected to immunoblotting with anti-EGFR antibody. Ten percent of each original total cell lysate was used as input quantification, using β-actin as loading control. All error bars show standard error for triplicate experiments. *, *P* < 0.05; **, *P* < 0.01; ***, *P* < 0.001; NS, not significant by Student t-test. **B.** Activation of Ras and the MEK/MAPK pathway in *MIIP*-HA−overexpressing or *MIIP*-knockdown H1299 cells. Numbers below the western blotting bands represents the quantification of gel densitometry. **C.** Cell proliferation was determined in MIIP-HA−overexpressing or MIIP-knockdown H1299 and A549 cells. Single-cell suspensions prepared from freshly selected cells positive for MIIP overexpression or MIIP knockdown were seeded in 6-well plates, and counted at indicated time points in triplicate. *, *P* < 0.05; **, *P* < 0.01; NS, not significant by repeated measures ANOVA.

We determined the EGF-binding activity of EGFR proteins from MIIP-HA−overexpressing and control H1299 cells. As shown in Figure 5A, the same amount of EGF-conjugated beads bound to more EGFR proteins in freshly selected MIIP-overexpressing H1299 cells, although the steady-state EGFR protein levels in these cells were about half those in control cells. This result indicates that the binding of EGFR and EGF in MIIP-HA−overexpressing H1299 cells was not impaired but increased, partially because of the cells' feedback regulation to maintain growth.

In H1299 cells cultured in normal medium containing FBS, we observed that EGFR maintained a steady level of Y1068 phosphorylation and downstream Ras and MEK/ERK activation. Phosphorylated EGFR and activated MEK/ERK were decreased in freshly selected MIIP-overexpressing H1299 cells, while the inverse effect was observed in MIIP-knockdown cells (Figure 5B). We noticed that the levels of total MEK and ERK proteins did not change in MIIP-overexpressing or knockdown cells, indicating that MIIP did not cause nonspecific protein decrease in the cells. The ratios of increased p-EGFR and decreased p-EGFR were close to the total-EGFR level changes in MIIP-knockdown and -overexpressing H1299 cells, respectively. This suggests that MIIP inhibits EGFR kinase activation not directly but through its downregulation of EGFR protein level.

MEK/ERK signaling is the primary pathway via which EGFR functions to promote cell proliferation. The activated ERKs translocate to the nucleus and transactivate transcription factors, changing gene expression to promote cell cycle progression [28]. As we had proven that MIIP decreases EGFR/Ras/MEK/ERK pathway activation via decrease of EGFR protein expression, we tested the growth of MIIP-overexpressing or knockdown lung cancer cells. Freshly selected H1299 cells overexpressing MIIP grew significantly more slowly than control cells, while MIIP-knockdown cells grew significantly more quickly (Figure 5C). A similar result was observed in MIIP-overexpressing A549 cells, but cell proliferation was not changed in sh*MIIP* A549 cells, probably because EGFR protein stability was not changed in this cell line by decreasing MIIP (Figure 1A). These results further demonstrate that MIIP attenuates lung cancer cell proliferation via its decrease of EGFR protein stability.

### MIIP protein expression correlates negatively with EGFR protein expression in NSCLCs and predicts patients' survivals

**Figure 6:**
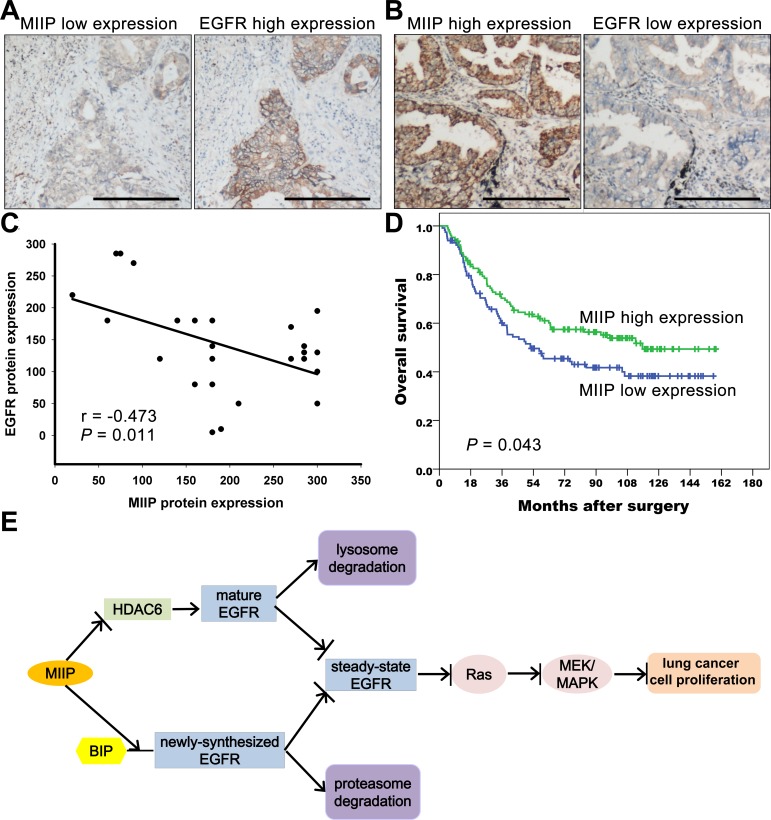
MIIP protein expression correlates negatively with EGFR protein expression in lung adenocarcinoma specimens and predicts patients' survivals **A.** Representative immunohistochemical staining of a lung adenocarcinoma case with low MIIP and high EGFR expression. (Scale bar: 40μM) **B.** Representative immunohistochemical staining of a lung adenocarcinoma case with high MIIP and low EGFR expression. (Scale bar: 40μM) **C.** Correlation between MIIP and EGFR protein expression in 28 lung adenocarcinoma specimens (Spearman's rank correlation analysis, r = −0.473, *P* = 0.011). **D.** Kaplan-Meier survival analysis of 234 Stage IA to IIIA lung adenocarcinoma patients with different MIIP expression levels (*P* = 0.043 by log-rank test). **E.** MIIP accelerates EGFR protein turnover, which results in the inhibition of lung cancer cell proliferation. MIIP enhances the binding between newly-synthesized EGFR protein and BIP, leads to the degradation of the nascent EGFR through the proteasome pathway. In addition, MIIP could increase the trafficking of mature EGFR into late endosomes and subsequent degradation in lysosome and decrease EGFR recycling back to cellular membrane by directly decreasing HDAC6 expression, one of the downstream targets of MIIP. The downregulation of steady-state EGFR by MIIP results in less activation of EGFR and downstream Ras/MEK/ERK pathway, which finally inhibits lung cancer cell proliferation.

To verify the *in vitro* results of negative correlation between MIIP and EGFR protein expression, we evaluated the expression level of MIIP and EGFR protein in 28 pairs of clinical adenocarcinoma NSCLC specimens by immunohistochemistry. Figure 6A and 6B showed two representative cases with low MIIP expression and high EGFR expression and vice versa. Spearman's correlation analysis demonstrated that the immunoreactivity score of MIIP was significantly inversely correlated with that of EGFR in adenocarcinoma NSCLC specimens. (Figure 6C, r = −0.473, *P* = 0.011). We next analyzed the expression of MIIP protein in a cohort of 243 Stage IA-IIIA adenocarcinoma NSCLC patients who underwent radical surgery. The positive staining of MIIP could be detected in 99.6% (242/243) adenocarcinoma NSCLC specimens. The median immunoreactivity score of MIIP expression in the tumor tissues was 180, and we defined a case with immunoreactivity score more than 180 as high expression, and otherwise low expression. According to this criterion, 47.7% (116/243) cases were classified as low expression, and 52.3% (127/243) high expression. There was no statistically significant association between MIIP expression level and patients' genders, ages, tumor differentiation, smoking history, or TNM stages (Chi-square test, *P* > 0.05, Table 1). By Kaplan-Meier analysis with log-rank test, patients with higher MIIP expression demonstrated longer overall survival compared with those with lower MIIP expression (Figure 6D, median 116.7 months vs. 52.3 months, *P* = 0.043). By Cox's proportional hazards regression model, MIIP expression [risk ratio (RR) 0.650, 95% confidence interval (CI) 0.454-0.930, *P* = 0.019], as well as pathological stage (RR 1.785, 95% CI 1.463-2.177, *P* < 0.001) and tumor differentiation grade (RR 1.521, 95% CI 1.062-2.178, *P* = 0.022) were selected as independent predictors of overall survival, but not other clinicopathological factors including patients' age, gender, or smoking history (*P* > 0.05).

**Table 1:** Expression of MIIP and clinicopathological characteristics of 234 surgically resected Stage IA to IIIA lung adenocarcinoma patients

Variables	MIIP expression in lung adenocarcinomas			
	Cases	Low, *n*(%)	High, *n* (%)	*P*^a^
Gender				0.560
Male	161	79 (49.1)	82 (50.9)	
Female	82	37 (45.1)	45 (54.9)	
Age (years)				0.542
<60^b^	116	53 (45.7)	63 (54.3)	
≥60	127	63 (49.6)	64 (50.4)	
Smoking history				0.970
Yes	126	60 (47.6)	66 (52.4)	
No	117	56 (47.9)	61 (52.1)	
Differentiation				0.664
Well and moderate	148	69 (46.6)	79 (53.4)	
Poor	95	47 (49.5)	48 (50.5)	
pTNM stage				0.475
Stage IA and IB	122	61 (50.0)	61 (50.0)	
Stage IIA and IIB	34	13 (38.2)	21 (61.8)	
Stage IIIA	87	42 (48.3)	45 (51.7)	

a Chi-square test.

b median age.

Thus, the results from clinical NSCLC samples were consistent with our finding from *in vitro* experiments that MIIP accelerated EGFR protein turnover and resulted in the inhibition of lung cancer cell proliferation. Taken all together, MIIP enhances the degradation of newly-synthesized and mature EGFR protein in proteasome and lysosome pathways, respectively. This leads to the downregulation of steady-state EGFR and less activation of EGFR and downstream Ras/MEK/ERK pathway, which inhibits lung cancer cell proliferation (Figure 6E).

## DISCUSSION

The *MIIP* gene is located on chromosome 1p36.22, which is one of the most frequently deleted regions in a wide spectrum of human cancers, including lung cancers [29]. It has been shown to exert tumor suppressor activity by inhibiting cell proliferation, invasion, and migration, and thus to play a critical role in cancer physiology. In the present study, we found that MIIP accelerates EGFR degradation via both proteasomal and lysosomal pathways. Furthermore, MIIP-promoted downregulation of EGFR inhibits downstream activation of Ras and blocks the MEK signal transduction pathway, resulting in inhibition of lung cancer cell proliferation. And higher MIIP protein expression predicts a favorable survival of adenocarcinoma NSCLC patients.

EGFR is highly expressed in a variety of human tumors, including NSCLC, and is implicated in tumor development and progression. It is known as a major regulator of cancer cell proliferation, migration, and anti-apoptosis by stimulating downstream signaling pathways after its activation. Either overexpression of wild-type EGFR or mutations in the kinase domain could lead to overactivation of the protein and its downstream pathway [30]. We observed that EGFR protein was decreased in lung cancer cells by MIIP overexpression and increased by MIIP knockdown. By labeling cells with ^35^S so as to observe the whole process of EGFR maturation and degradation, we found that MIIP-promoted EGFR degradation could happen in two stages during EGFR turnover, in the newly synthesized and in the mature EGFR, whose degradation is mediated by proteasome and lysosome, respectively.

Previous research has proved that disrupting the glycosylation of EGFR reduces both cellular protein levels and receptor activity in tumor cells through retention of the receptors in the ER/Golgi compartments [31]. In our MIIP-overexpressing lung cancer cells, EGFR level decreased as early as the appearance of the semiglycosylated 160-kD EGFR peptides. This decrease could be reversed by proteasome inhibitor lactacystin, suggesting that a proteasome pathway is involved in this ERAD process. Furthermore, stronger binding between newly synthesized EGFR and BIP was observed in MIIP-overexpressing cells, although change in BIP expression was not observed. BIP has been postulated to sequentially bind, release, and rebind newly synthesized proteins until the proteins fold correctly and no longer accessible to BIP. Proteins that do not fold correctly remain bound to BIP and are not transported further along the secretory pathway [8]. Cai *et al.* [32] found that BIP formed a stable complex with the underglycosylated EGFR under stress conditions and inhibited EGFR's translocation to the cell surface. The enhanced binding we observed between BIP and EGFR in MIIP-overexpressing cells suggests that there may be some folding defects of newly synthesized EGFR in such cells, which might lead to degradation of these misfolded EGFR proteins via ERAD. Therefore, it is possible that MIIP destroys nascent EGFR peptide folding and/or glycosylation via some mechanism we are not sure of, resulting in degradation of the nascent EGFR in the ER through the proteasome pathway. Further studies are needed to specify how MIIP affects these ER chaperones' function in maturation of EGFR or other secretory proteins.

Besides the proteasome-mediated degradation of nascent EGFR, MIIP-overexpressing cells showed increased degradation of mature EGFR in the late phase of our chase experiment, from chase 4 h to 10 h. As demonstrated in Figure 4A, this process was significantly inhibited by chloroquine, indicating involvement of the lysosome. At steady-state cell growth conditions, the distribution of EGFR is determined by the ratio of the internalization and recycling rates, resulting in predominant localization of EGFR at the cell surface and in a small endosomal pool [13, 24–26]. As our immunofluorescence results shown, more EGFR were co-localized with the late endosome/lysosome marker LAMP1 but less with the recycling marker Rab11 in MIIP-HA−overexpressing cells after 5min EGF treatment and 30min chase, suggesting a requirement for MIIP in the lysosomal degradation of EGFR by ensuring the trafficking of activated EGFR into late endosomes/lysosome and decreasing EGFR recycling back to cellular membrane.

Intensive studies have been conducted on EGFR endocytosis and its intracellular transport. The transport of endocytic contents is dependent on microtubules, and microtubules can promote fusion of endocytic vesicles [33]. Acetylation of α-tubulin, one component of αβ-tubulin heterodimers, could lead to increased microtubule stability [34] and affects microtubule-based functions [35], including intracellular trafficking. Gao *et al.* [18] found that transition of EGFR from early to late endosome and final degradation could be accelerated by HDAC6, a microtubule-associated deacetylase. Our previous research proved that MIIP could bind directly to the catalytic domains of HDAC6, resulting in decreased HDAC6 activity and resultant increased levels of acetylated tubulin [17]. Therefore, MIIP might increase the fraction of shuttling EGFR targeted to the lysosomal compartment to exert its role in enhancing degradation of mature EGFR. In addition, a recent genome-wide siRNA screens identified the involvement of MIIP in Nef-induced downregulation of MHC-1 [36]. Nef, a HIV-1 encoded protein, could induce MHC-1 to enter the endocytic trafficking and be degraded finally in lysosomes, which would aid in HIV-infection. This also suggests the important role of MIIP in endocytic trafficking and degradation of membrane proteins, although the specific role of MIIP in HIV-infection is not clear.

The negative correlation between MIIP and EGFR protein expression observed *in vitro* was verified in adenocarcinoma NSCLC specimens. More importantly, MIIP expression was identified as an independent predictor for overall survival of adenocarcinoma NSCLC patients. The better survival of patients with high MIIP expression may be attributable to the intrinsic characteristics of the tumor cells partly determined by MIIP, although no significant correlation was observed between MIIP expression and patients' pathological stage or differentiation.

In this study, we found that MIIP accelerates EGFR protein turnover via proteasomal degradation in the ER and then by the lysosomal pathway after its entry into endocytic trafficking. The process of EGFR downregulation and degradation could be a regulatory mechanism of MIIP that controls the intensity and duration of EGFR-mediated signaling. Our results show that downregulation of EGFR by MIIP suppresses cancer cell growth significantly. This reveals a new mechanism through which MIIP inhibits cancer cell proliferation. Disruption of newly synthesized EGFR in the ER might be an alternative method for reducing EGFR signaling in addition to tyrosine kinase inhibitor therapy targeting mature EGFR at the membrane [37]. It is possible, moreover, that MIIP might accelerate ER-associated degradation of other membrane proteins that undergo common synthetic and folding steps in the ER. This might provide a therapeutic strategy for disrupting signaling from multiple transmembrane receptors. In summary, we have found that tumor suppressor MIIP promotes EGFR degradation via both the proteasomal and lysosomal pathways, resulting in downregulation of EGFR downstream signaling and ultimately in inhibition of lung cancer cell growth. Determination of MIIP protein expression could serve as a prognostic marker of NSCLCs.

## MATERIALS AND METHODS

### Reagents and antibodies

Chloroquine, brefeldin A, and lactacystin were purchased from Sigma-Aldrich Corporation (St. Louis, MO). Protein G Plus-Agarose was purchased from EMD Biosciences (San Diego, CA). Antibody against human MIIP for western blotting was generated as described previously [38]. Antibodies against human MIIP for immunohistochemistry were purchased from Sigma-Aldrich. Anti-EGFR, anti-EEA1, anti-Rab11, anti-LAMP1, anti-ubiquitin and anti-HA (to detect the recombinant MIIP-HA protein) were purchased from Santa Cruz Biotechnology (Santa Cruz, CA), and anti-phospho-EGFR (Y1068), phospho-p44/42 MAPK (Erk1/2) (Thr202/Tyr204), p44/42 MAPK (Erk1/2), phospho-MEK1/2 (Ser217/221), and MEK1/2 were purchased from Cell Signaling Technology (Beverly, MA). EasyTag EXPRESS^35^S Protein Labeling Mix was purchased from PerkinElmer Life Sciences (Waltham, MA).

### Cell culture

The NSCLC lung cancer cell lines H1299, A549, and H322 were purchased from American Type Culture Collection (Manassas, VA) and cultured in RPMI 1640 medium supplemented with 10% FBS, 100 U/ml penicillin, and 100 U/ml streptomycin in a humidified incubator containing 5% CO_2_ at 37°C.

### Transfection

The *C*-terminal HA-tagged wild-type MIIP construct was custom-made by OriGene Technologies, Inc. (Rockville, MD) and based on the Precision Shuttle vector pCMV6-AC-HA (PS100004). *MIIP* and control shRNA plasmids were purchased from Santa Cruz Biotechnology.

Plasmid DNA was transfected by using Lipofectamine 2000 reagent according to the manufacturer's protocol (Life Technologies, Carlsbad, CA). Briefly, cells were subcultured on dishes or multiwell plates at a density of 90%. Transfections were performed the next day using 20 μg of plasmid DNA/100-mm dish. Cells were collected 2 days after transfection for preparation of protein lysate or RNA for western blotting or real-time RT-PCR, respectively.

Freshly selected H1299 and A549 cells stably transfected with *MIIP* were obtained after 2 weeks selection by G418 (600 mg/ml). For generation of stable *MIIP*-knockdown cells, sh*MIIP*-transfected H1299 and A549 cells were selected in the presence of puromycin (0.5 mg/ml) for 2 weeks.

### Real-time RT-PCR

RNA samples for real-time RT-PCR were prepared from aliquots of H1299 cells stably transfected with *MIIP* or sh*MIIP* and their matched control cells. Real-time RT-PCR was performed on the ABI Prism 7900 (Life Technologies, Foster City, CA) using the commercially available TaqMan gene expression assay for human EGFR and housekeeping gene human β-actin as control. The 7900 Sequence Detection System 2.3 software was used to determine the fold-change for EGFR using the δδCt method with 95% confidence.

### Metabolic labeling

Metabolic labeling of cultured cells was performed by pulse-chase experiment. Freshly *MIIP*-transfected and control H1299 cells were subcultured into 60-mm or 100-mm dishes at a cell density of 80%. The next day, cells were starved for 1 h by incubation in methionine-free medium and then labeled for the periods indicated in the figure legends with 10 ml of EasyTag EXPRESS^35^S Protein Labeling Mix (11 mCi/ml)/60-mm dish. In experiments where a chase followed, the isotope-containing medium was replaced after 40 min labeling by complete medium containing 5% FBS and 0.2 mM excess unlabeled L-methionine. In experiments without a following chase, cells were collected after 10, 20, 40, or 60 min labeling. To evaluate the influence of lactacystin, chloroquine, or brefeldin A, cells were preincubated with the concentration of these compounds indicated in the figure legends for 60 min prior to methionine starvation. The concentrations of these drugs were maintained at the same levels during starvation, pulse, and chase periods.

### Immunoprecipitation

Cells were subjected to lysis using a solution of 50 mM Tris-HCl (pH7.5), 0.15 M NaCl, and 1% Nonidet P-40 containing Halt protease inhibitor cocktail (Thermo Scientific, Rockford, IL). Following centrifugation at 4°C at 14000*g* for 20 min, the supernatant was incubated overnight with appropriate primary antibody. Protein G Plus-Agarose was then used to remove the complexes formed. After four washings of the beads with lysis buffer, the attached complexes were dissolved in electrophoresis sample buffer.

### SDS-PAGE and immunoblotting

The immunoprecipitates and total cell lysates were subjected to electrophoresis on 8% or 10% acrylamide gels. After electrophoresis of immunoprecipitates, gels were dried and analyzed by electronic autoradiography. For western blotting, proteins in the gels were transferred to nitrocellulose filters (Amersham, Piscataway, NJ) and probed with the primary antibodies specified in the figure legends after blocking with 5% dried milk and HRP-conjugated anti-rabbit or anti-mouse IgG (Amersham). The antigen-antibody complexes were visualized by SuperSignal Chemiluminescent reagents (Thermo Scientific). The specific intensity of each protein band on X-ray film was measured by IMAGE J software from the National Institutes of Health and expressed as a ratio of the optical density band of each protein to that of β-actin.

### Immunofluorescence analysis

Freshly MIIP-transfected and control H1299 cells were cultured on coverslips overnight. After adherence, cells were serum-starved in serum-free medium for 24h, and then EGF (10ng/ml) made in 37°C pre-warmed serum-free medium was added to cells. EGF treatment lasted for 5min. Immediately, the cells were fixed for 20min at room temperature in 4% paraformaldehyde in PBS or chased in serum-free medium without EGF at 37°C for 30min and followed by fixation in paraformaldehyde. For immunofluorescence staining, the cells were permeabilized for 20min at room temperature in 0.25% Triton X-100 in PBS, and blocked for 1h at room temperature in 1% BSA in PBS. Slides were then incubated overnight at 4°C with indicated antibodies. Then slides were stained for 2h at 4°C with the corresponding FITC or Texas Red-conjugated secondary antibodies (Santa Cruz) and mounted with mounting medium with anti-fading agent (Electron Microscopy Sciences, Hatfield, PA). Microscopic images were obtained with an Olympus BX51 immunofluorescence microscope. Signal intensity and colocalization were measured with ImageJ software. Magnification was ×1000.

### EGF-binding activity assay

EGF-conjugated beads were prepared by immunoprecipitating EGF from EGF solution (Cell Signaling Technology) by incubating with anti-EGF polyclonal antibody from rabbit conjugated to Protein G Plus-Agarose overnight. Stable MIIP-transfected and control H1299 cells were subjected to lysis in Nonidet P-40 (1%) buffer containing protease inhibitors with a protein concentration of 2 mg/ml. Samples (90% of each of the total cell lysates) were incubated at 4°C overnight with EGF-conjugated beads. EGFR bound to the EGF-conjugated beads was pulled down and subjected to immunoblotting with anti-EGFR antibody. Ten percent of each original total cell lysate was used as input quantification, using β-actin as loading control.

### Cell proliferation assays

Single-cell suspensions were prepared from freshly selected cells positive for MIIP overexpression or MIIP knockdown in exponential growth phase. Aliquots of 10,000 cells were seeded in 6-well plates. Three wells were used for each determination, and after staining with 0.4% trypan blue (Sigma-Aldrich), cells in each well were counted three times every 24 h for 5 days.

### Determination of Ras activation status

Lysates of stably *MIIP*-transfected, *MIIP*-knockdown, or control H1299 cells were incubated with glutathione agarose-conjugated GST-fused recombinant Raf-1 protein corresponding to the human Ras-binding domain (Millipore, Temecula, CA). GTPγS and GDP were added to cell lysates as positive and negative controls, respectively. The activated form of Ras, GTP-Ras, was pulled down by the Raf-1 Ras-binding domain agarose and subjected to immunoblotting with anti-pan-Ras antibody.

### Tumor specimens

The Ethics Committee of the Sun Yat-Sen University Cancer Center approved the use of tumor specimens in this study. A total of 234 patients with Stage IA to IIIA adenocarcinoma NSCLC who underwent radical surgery with formalin-fixed and paraffin-embedded tumor samples available at Sun Yat-sen University Cancer Center from 2001 to 2006 were retrospectively enrolled in this study. The disease stages of all patients were classified or reclassified according to the UICC 2009 TNM staging system. No patient underwent neoadjuvant chemoradiotherapy before surgery. The survival status of all the recruited patients were verified in February 2015, and overall survival was defined as the time surgery to death, censoring patients who were still alive at the date of last follow-up

### Immunohistochemistry

MIIP and EGFR protein staining were performed on 234 cases and randomly selected 28 cases, respectively. Sections (4 μm) of each tissue block were cut and placed on glass slides. After rehydration through graded alcohols, endogenous peroxidase activity of the slides was blocked with 3% hydrogen peroxide for 10min. For antigen retrieval, tissue slides were boiled in Tris-EDTA buffer (pH 8.0) in a pressure cooker for 2.5 min. Nonspecific binding was blocked with 10% normal goat serum for 10min. The tissue slides were incubated with rabbit polyclonal anti-MIIP (HPA044948, 1: 50 dilution) or anti-EGFR (sc-03, 1:1000) at 4°C overnight in a moist chamber. Immunoperoxidase staining was carried out using the Envision Detection Systems (DAKO, Glostrup, Denmark) according to the manual. Briefly, after primary antibody incubation, the slides were incubated with a HRP conjugate peroxidase working solution for 30min at 37°C. Finally, the slides were reacted with 3,3′-diaminobenzidine as a chromogen substrate, and counterstained using hematoxylin (Sigma). A negative control was obtained by replacing the primary antibody with a normal rabbit IgG.

A pathologist blinded to the clinicopathological information performed the evaluation of MIIP expression. MIIP cytoplasmic expression was scored by using a semi-quantitative system. The staining intensity (0 = no intensity, 1= weak, 2 = moderate, and 3 = strong) and proportion of positive lung cancer cells of each slide with at least 200 cells counted were recorded individually. Immunoreactivity score was calculated by multiplying the percentage of positive cells by the score of signal intensity, yielding values ranging from 0 to 300 [39].

### Statistical analysis

Calculations of mean value ± standard error were based on three independent experiments. Data analysis was carried out using the SPSS 19.0 statistics software package (IBM, Armonk, NY). Continuous variables were analyzed using the Student *t*-test. Repeated measures ANOVA analysis was performed to assess the statistical significance of the means of band intensities at different time points between MIIP-HA−overexpressing and control cells in pulse-chase experiments. The relationship between MIIP and EGFR expression was explored by Spearman's rank correlation. The correlation between MIIP expression and clinicopathological parameters was analyzed by Chi-square test. Survival curves were analyzed by the Kaplan-Meier method and log-rank test. To determine independent factors significantly related to the prognosis, multivariate analysis was performed using Cox's proportional hazards regression model with a forward stepwise procedure (the entry and removal probabilities were 0.05 and 0.10, respectively) and conditional likelihood ratio test. A two-tailed *P*-value of < 0.05 was considered statistically significant.
